# Atopic dermatitis epidemiology and unmet need in the United Kingdom

**DOI:** 10.1080/09546634.2019.1655137

**Published:** 2019-10-21

**Authors:** Michael J. Cork, Simon G. Danby, Graham S. Ogg

**Affiliations:** a Sheffield Dermatology Research, Department of Infection, Immunity & Cardiovascular Disease, Faculty of Medicine, Dentistry & Health, The University of Sheffield, Sheffield, UK;; b Sheffield Children’s Hospital and Sheffield Teaching Hospitals Clinical Research Facilities, Sheffield, UK;; c MRC Human Immunology Unit, NIHR Biomedical Research Centre, Weatherall Institute of Molecular Medicine, University of Oxford, Oxford, UK

**Keywords:** Atopic dermatitis, disease burden, financial cost, unmet need

## Abstract

Atopic dermatitis (AD), also known as atopic eczema, is a chronic inflammatory skin condition associated with a significant health-related and socioeconomic burden, and is characterized by intense itch, disruption of the skin barrier, and upregulation of type 2-mediated immune responses. The United Kingdom (UK) has a high prevalence of AD, affecting 11–20% of children and 5–10% of adults. Approximately 2% of all cases of childhood AD in the UK are severe. Despite this, most AD treatments are performed at home, with little contact with healthcare providers or services. Here, we discuss the course of AD, treatment practices, and unmet need in the UK. Although the underlying etiology of the disease is still emerging, AD is currently attributed to skin barrier dysfunction and altered inflammatory responses. Management of AD focuses on avoiding triggers, improving skin hydration, managing exacerbating factors, and reducing inflammation through topical and systemic immunosuppressants. However, there is a significant unmet need to improve the overall management of AD and help patients gain control of their disease through safe and effective treatments. Approaches that target individual inflammatory pathways (e.g. dupilumab, anti-interleukin (IL)-4 receptor α) are emerging and likely to provide further therapeutic opportunities for patient benefit.

## Introduction

Atopic dermatitis (AD), also known as atopic eczema, is a chronic inflammatory skin condition associated with epithelial, immune, and environmental factors. It is characterized by intense itch, disruption of the skin barrier, and upregulation of type 2-mediated immune responses in the skin (1–4).

As a disease, AD is characterized by early age of onset, with approximately 60% of AD cases in the UK diagnosed in the first year of life (5). Prevalence of AD decreases with age, with 30% of 4-year-olds, 11–20% of school-aged children, and 5–10% of adults diagnosed with AD (6,7). Data from the International Study of Asthma and Allergies in Childhood (ISAAC) and other studies (8–10) showed that among children within a general practitioner (GP) setting or within the general population, the annual AD prevalence varies between age groups, and highlighted differences between self-reported prevalence of AD in the open population compared with physician-diagnosed disease in general practice (10).

Severity of AD can be assessed objectively in a standardized manner using the SCORing AD (SCORAD) index. Higher numbers indicate greater severity, and the scale ranges from 0 to 103 (11). Approximately 18% of all cases of childhood AD in the UK are moderate (as defined by Ben-Gashir et al., SCORAD = 16–40) and 2% severe (SCORAD ≥40) (4). The odds of having severe AD are twice as great for children with AD onset during the first year of life (4). Moderate-to-severe AD can not only impact a child’s physical development but can also have psychological sequelae, placing a substantial burden on parents and carers (12,13). Fortunately, diagnosis of AD is typically accurate in secondary care – a review of dermatology cases over a 25-year period in Scotland identified AD with 97% accuracy (14). However, given that most treatments are performed at home with little GP or hospital services involvement, there remain significant challenges with associated health-related and socioeconomic burdens (12,13).

### The individual financial cost for AD

A recent study in the United States of America (USA) investigated the costs, stratified by severity, for adults with moderate-to-severe AD inadequately controlled with topical therapy, or for whom topical therapies were medically inadvisable (15). The average lifetime cost for usual care was 271,356 USD for patients with moderate AD and 271,579 USD for patients with severe AD (15). Because of the differences between healthcare systems in the UK and USA, however, it is difficult to correlate these lifetime costs with the financial burden in the UK.

A study comprised of adults with AD from nine different European countries including the UK found that out-of-pocket costs accounted for about 900 EUR (∼800 GBP) per year, including moisturizers and emollients, medications, travel expenses, and other costs. Additionally, many patients had extra costs related to everyday expenses, such as the purchase of extra or special cleaning products or washing powder (laundry detergent), bedding, or clothing that otherwise would not be purchased (16).

On top of direct costs, indirect costs associated with AD include disruption of employment (time off work, reduction in employment, and loss of productivity). In the European study, 26% of patients missed 6–10 days at work within the last year due to their AD, and over half missed 1–5 days. Patients with moderate-to-severe AD were more likely to miss work (16). In addition to patients themselves, indirect costs affect carers as well. For example, mothers of children with AD were found to be less likely to take up outside employment or to pursue leisure activities compared with mothers of children without AD (13,17,18).

It is unclear how the financial burden of AD impacts treatment compliance in patients (19–22). The large-scale International Study of Life with Atopic Eczema (ISOLATE) investigated the effect of AD on patients’ lives and society; it examined how patients and their carers coped with AD and how well they believed their disease was being controlled. Most of the patients in the study were prescribed reactive topical corticosteroid (TCS)-based AD therapies (20). Although effective, concerns over TCSs led to compliance issues and treatment delays or restrictions, resulting in 39% of participants using TCSs less frequently and for shorter periods than was recommended, and 66% using TCSs only as a last resort. The results of ISOLATE highlight AD as an undertreated disease, which, despite the availability of effective therapies, has considerable, yet often avoidable, adverse effects on patients, their carers, and society – including socioeconomic costs (e.g. unemployment, lost productivity, and an impact on schoolwork, learning, and academic performance among younger patients) (20).

An audit commissioned by the British Association of Dermatologists provided data on the national service outcomes of 235 patients with AD in secondary care in the UK (23). The audit gathered information from patients using pre- and post-consultation questionnaires in 29 hospital dermatology departments randomly selected from 187 centers. The outcomes measured were quality of life (QoL), sleep improvement, improvement in the worst aspect of AD, and the ability to return to work or school (23), and were based on audit standards established by the British Association of Dermatologists and the Research Unit of the Royal College of Physicians (24).

An improvement in QoL (>25%) was reported in 49% of adults and 44% of children, and improved sleep was reported in 44% of adults and 47% of children; however, these results fell short of the working standards of 60% for QoL and 70% for improved sleep. Further, an improvement in the worst aspect of AD was reported in 61% of adults and 59% of children, but that too fell short of the working standard of 80%. Although 87.5% of children returned to school within 6 weeks and met the working standard of 80%, only 70% of adults returned to work, again falling short of the working standard (23). It should be taken into account that only a small percentage of AD patients are referred to dermatologists for care in the UK (4% of children aged 1–5) (19) therefore poor outcomes may reflect selection bias for patients with severe and recalcitrant disease. Nevertheless, these studies demonstrate a significant need to improve the management of AD and help patients gain control of their disease.

### Pathogenesis and course of AD

Although the underlying etiology of AD is not fully known, it is believed to be attributable to complex, yet interrelated, biologic pathways, including dysfunction of the skin barrier and altered innate or adaptive immune responses (25). There is increasing evidence that disruption of the skin barrier function and atopy affect one another reciprocally, ‘driving’ the progress of AD (26–28).

The stratum corneum (SC) is composed of corneocytes, terminally differentiated enucleated keratinocytes that are densely packed with lipids and proteins (29). Filaggrin contributes to SC function through many roles, including keratin cross-linking, hydration, and pH modulation (30–34). Filaggrin is naturally broken down in the SC into several compounds that are the constituents of natural moisturizing factor (NMF) (31,33,35). NMF is essential for optimal SC hydration, desquamation, plasticity, and acidity, and it provides the optimal environment for commensal microorganisms colonizing the skin (32,33,35,36). Disruption of the healthy epidermal microbiome can be associated with skin disorders or infections by potentially pathogenic bacteria such as *Staphylococcus aureus* (37–39).

Many genetic factors influence the integrity of the skin in AD, including mutations in genes encoding structural proteins, such as filaggrin (25,40). Loss-of-function mutations in the gene encoding filaggrin (*FLG*) have been associated with early-onset, severe, and long-lasting AD, and are considered to be the most significant genetic risk factor for developing the disease (25,26). Mutations in proteases and protease inhibitors also play an important role in AD, leading to altered desquamation and defects in the skin barrier (40–42). These and other immunological genetic factors (43–45) are thought to provide the underlying susceptibility that may predispose individuals to develop AD (25,41,42,46–48).

Environmental trigger factors are believed to play an important role in the progression of disease and development of AD (49). Data obtained (at age 7, 11, and 16 years) from 828 children born in 1958 showed a marked and statistically significant geographical variation in AD prevalence. The highest risk was associated with London and the South-East, North Midlands, Eastern, and Southern regions of the UK (49). In other studies, urban areas have been shown to have a higher risk of severe disease than rural areas (4,50). These regions may be associated with environmental factors such as temperature and humidity, allergen exposure, microbial exposure, pollution, and lifestyle factors (51,52). Itch is a key symptom of AD and promotes physical disruption of the skin barrier (53), which can promote the penetration of allergens such as the house dust mite *Dermatophagoides* protease (Der p1). Such proteases have been linked directly to the degradation of the skin barrier (54–56). Other environmental factors known to impact AD include water hardness and contaminants in water (57), soaps and detergents (58,59), and prolonged use of TCSs (60,61).

AD is a product of interplay between such environmental factors and genetic susceptibility. The loss-of-function *FLG* mutation results in decreased levels of filaggrin and, consequently, reduced NMF. Low levels of NMF increase transepidermal water loss and elevate SC pH levels (33,35). This altered skin environment can lead to *S. aureus* infection (62,63), which in turn leads to skin inflammation and systemic immunoglobulin (Ig) E sensitization (64–66). *S. aureus* can damage the skin barrier directly and secrete exotoxins that can activate an immune response to allergens penetrating the skin barrier (64,65,67–69). For example, one *S. aureus* exotoxin functions as an adjuvant to promote the inflammatory response to Der p1 (70).

The penetration of allergens through the defective skin barrier results in interaction with local immune cells and in the release of AD-related pro-inflammatory cytokines (27,54,71–78).

**Figure 1.**
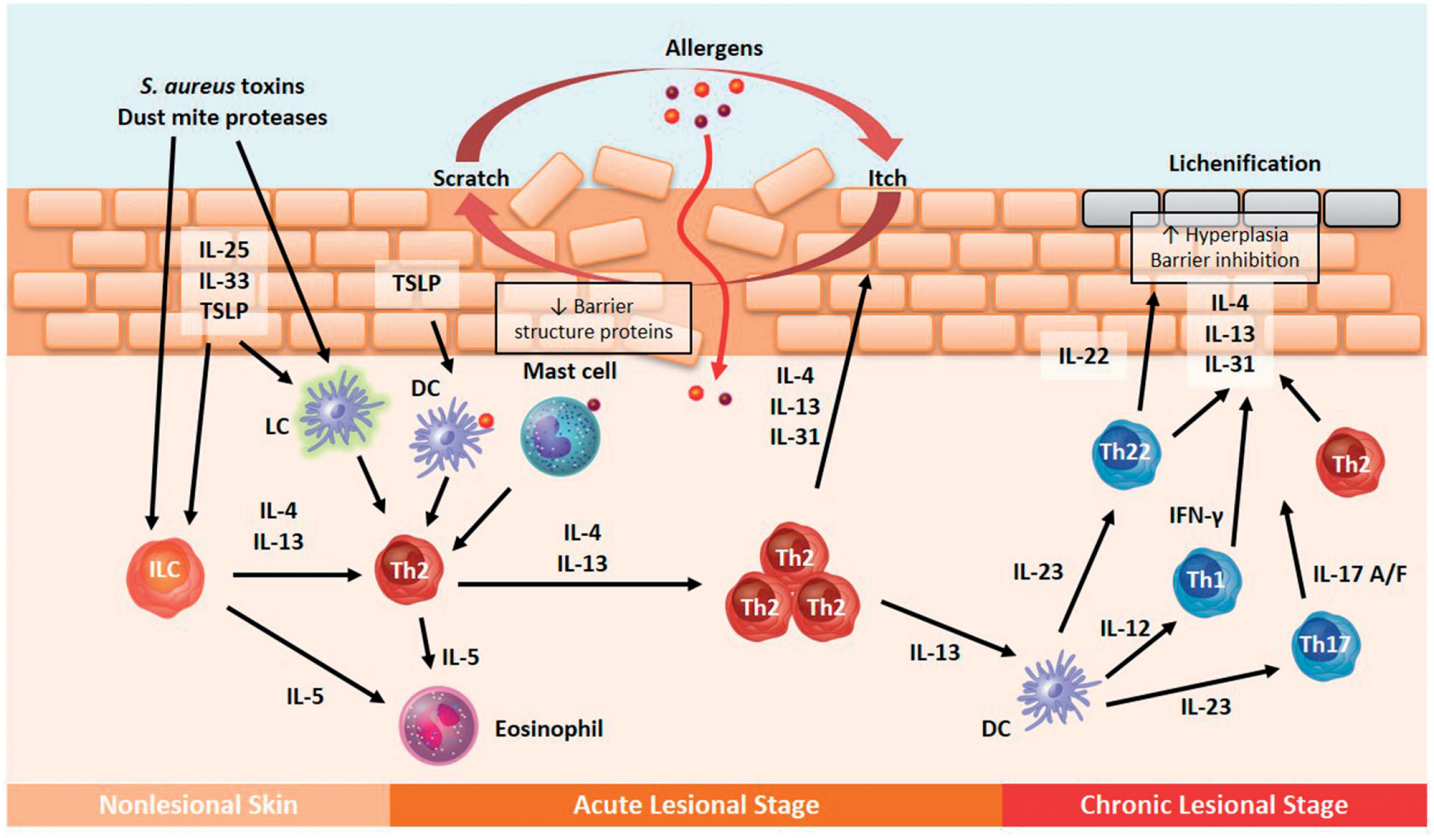
Skin barrier dysfunction and immune response in atopic dermatitis (AD). DC: dendritic cell; IFN-γ: interferon gamma; ILC: innate lymphoid cell; IL: interleukin; IL-17 A/F: IL-17 A/F homodimer or heterodimer; LC: Langerhans cell; Th1: T helper type 1 cell; Th17: T helper type 17 cell; Th2: T helper type 2 cell; Th22: T helper type 22 cell; TSLP: thymic stromal lymphopoietin.

During the initial or acute phase of AD, a type 2 (including innate lymphoid cells [ILCs] and T helper type 2 cells [Th2]) immune response characterized by interleukin (IL)-4, IL-13, and IL-5 predominates (Figure 1) (79–81). This may, in part, be related to the release from keratinocytes of type 2-driving alarmins (IL-25, IL-33, and thymic stromal lymphopoietin [TSLP]). In the chronic phase, a mixed response involving Th1, Th17, and Th22 immune cells can be observed (74–78,82,83).

Lesional skin biopsies from patients with acute and chronic AD are enriched for the type 2 cytokines IL-4, IL-5, IL-13, IL-31, and IL-33 (75,79–81). The IL-4 and IL-13 cytokines are critical for further type 2 polarization and the development of AD (80,84–87). IL-5 is produced by Th2 cells and other cells and promotes eosinophilic inflammation in atopic diseases (88). IL-31, primarily produced by Th2 cells and mast cells in response to antimicrobial peptides, is significantly increased in AD, and it has been implicated in the regulation of itch (53,89). IL-33 activates Th2 cells, ILCs, mast cells, neutrophils, and eosinophils in response to allergen or *S. aureus* exotoxin exposure and other triggers (77,78,90). Recently, it was shown that house dust mite-derived phospholipases act on the skin to produce antigenic neolipids that are presented by CD1a for recognition by T cells. The production of type 2 cytokines by ILCs, peptide-specific major histocompatibility complex-restricted T cells, and lipid-specific CD1a-reactive T cells supports the generation of allergen-specific IgE (28,91). Type 2 cytokines have also been shown to contribute to the skin barrier dysfunction by modulating the expression of structural proteins and antimicrobial peptides – key to maintaining the skin integrity – and thereby facilitating allergen penetration through the skin (Figure 1) (27,92). Both increased allergen-specific IgE presentation and allergen penetration through the skin barrier potentiate the inflammatory response.

The generation of IgE antibodies and skin-derived TSLP is associated with the development of other atopic disorders, including asthma, allergic rhinitis, and/or food allergies (93–97). Additionally, AD has recently been shown to be associated with non-atopic disorders, including cardiovascular disease (98,99) and some forms of cancer (100). The relation of localized skin disorders with systemic disease represents one of the largest challenges for treating AD and associated morbidity (101). Early and effective management of AD may therefore have effects beyond the skin.

### AD treatment in the UK

The aim of the guidance and information available on skin conditions provided by the UK's National Health Service (NHS) is to facilitate a whole system-integrated approach for people with AD that ensures timely access, high-quality care (close to home, where applicable), and value for money (102). In England and Wales, the NHS directive uses the standards set for patient care by the National Institute for Health and Care Excellence (NICE). The NICE guidelines cover the diagnosis and management of AD in children and adults to improve care and QoL, and to decrease the physical severity of their disease (103,104).

The management of AD in the UK occurs predominantly in the primary care setting, and current treatment options include approaches intended to protect the skin barrier (e.g. emollients [leave-on and wash], medicated bandages) or reduce inflammation (TCSs, topical calcineurin inhibitors [TCIs], broad immunosuppressants, biologics). Also significant in the management of AD is the identification, avoidance, and treatment of exacerbating environmental factors. As discussed above, disruptions in the skin barrier result in greater exposure to allergens, and avoiding such allergens can play a role in the management of AD. For children under the age of 12 years, a stepwise approach should be taken to manage the disease, with the potency of the medications adapted to the severity of the disease and the anatomical site of application (Table 1) (103).

**Table 1.** Holistic assessment of atopic dermatitis (AD) and treatment options for children under the age of 12 years (103).

	Skin/physical severity	Impact on quality of life and psychosocial well-being	Stepped approach to treatment
Clear	Normal skin, no evidence of active eczema	No impact on quality of life	
Mild	Areas of dry skin, infrequent itching (with/without redness)	Little impact on everyday activities, sleep, and psychosocial well-being	Emollients, mild potency topical corticosteroids (TCSs)
Moderate	Areas of dry skin, frequent itching, redness (with/without excoriation and localized skin thickening)	Moderate impact on everyday activities and psychosocial well-being, frequently disturbed sleep	Emollients, moderate potency TCSs, topical calcineurin inhibitors (TCIs), bandages and dressings
Severe	Widespread areas of dry skin, incessant itching (with/without excoriation, extensive skin thickening, bleeding, oozing, cracking, alteration of pigmentation)	Severe limitation of everyday activities and psychosocial functioning, nightly loss of sleep	Emollients, potent TCSs, TCIs, bandages and dressings, phototherapy, systemic therapy

Adapted from NICE, 2007 (103).

According to the NICE guidelines, the treatment options for children with AD should be tailored to meet the needs of the patient. Emollients such as creams, ointments, sprays, lotions, gels, and bath additives are considered first-line therapies and are selected by the patient (103,105). Emollients are products that contain various moisturizing components that improve symptoms, including humectants (hygroscopic substances that attract water) and non-physiologic lipids. The lipids provide an artificial protective layer over the surface of the skin that aids water retention and transiently improves skin barrier function. Emollients can help soften skin texture and help relieve the pruritus (itch) caused by excessive dryness (106), and some may even reduce the need for TCSs (107). Simple emollients are tolerated in children as young as 6 months (103,108).

The accepted best practice for emollient therapy recommends consistent and liberal use of emollients and skin protectants. Recent evidence suggests that not all emollients for the protection and maintenance of the skin barrier are the same, with some displaying additional physiological effects on the skin and others having adverse effects (109–112). For example, some emollients contain surfactants and emulsifying agents (such as sodium lauryl sulfate) that not only disrupt the epidermal barrier function (113,114) but can also irritate the skin and induce an immune response (58,115). In contrast, other emollients appear to delay the onset of flares and may even help prevent the primary emergence of AD (116,117). As such, many uncertainties still remain regarding the use of emollients, including which emollient to use and how much (118).

Bathing, by soaking in lukewarm water with emollients (and possibly short-term/intermittent antimicrobials), offers an opportunity to improve skin hydration, provides symptomatic relief of AD symptoms, and has an antipruritic effect (103,106). However, bathing can also cause dryness, especially if a harsh detergent is used. Therefore, non-soap-based cleansers and mild synthetic detergents (pH of 5.5–6.0) that protect the skin’s acid mantle are recommended for patients with AD (105).

While treating flares with TCSs can offer rapid and effective relief from symptoms, their long-term use carries potential safety concerns, such as cutaneous adverse events and possible systemic side effects (119). Over the years, however, these concerns have escalated into phobias (120), particularly among parents of pediatric AD patients (121–123). These phobias led to treatment noncompliance (120) and ultimately reduced disease control, which increased morbidity and the burden of the disease. ‘Corticophobia’ might also explain, at least partially, why patients often delay treatment of flares, resulting in the disease needlessly going untreated for extended periods. The introduction of a non-steroidal treatment option for patients with AD – TCIs (or topical immunomodulators) – is thus intended to complement the existing treatment choices and overcome the negatives associated with TCS therapy (103,124).

TCSs have been the mainstay of AD treatment for over 40 years. When a patient with AD first applies one of the more potent variants, the benefit is often rapid and apparent. However, increasing the potency of the preparation in response to tachyphylaxis (drug tolerance) (125) may lead to local adverse events. Furthermore, the side effects of persistent daily applications of a potent TCS can be unfavorable. As discussed above, prolonged use of TCSs can potentially damage the skin barrier, resulting in thinning of the skin, telangiectasia, or striae distensae (60,61,125). The potency of a TCS is partly determined by the amount of vasoconstriction produced and the degree to which it inhibits inflammation. Thus, a mild TCS can be used to treat a mild AD flare. The TCIs, tacrolimus and pimecrolimus, are not recommended as first-line therapy for AD in England and Wales (103,104). TCIs do not damage the skin barrier and are therefore particularly useful on skin sites with a thin skin barrier such as the face and flexures, which are most vulnerable to the adverse effects of TCSs (126). TCIs should be used only in the absence of clinical infections. For the use of both TCSs and TCIs, maintenance treatment twice per week can be helpful in reducing the frequency and severity of flares, although TCIs are preferable because of their positive effects on the skin barrier (103,126). The correct use of all topical therapies should be demonstrated by specialist dermatology nurses and care plans should be provided as part of an intensive educational package (103,104). Phototherapy may also be used in patients whose medical, physical, and/or psychological states are greatly affected by their AD (103). Narrowband ultraviolet B (UVB) light is the most common form of phototherapy because of its relative efficacy, availability, and provider comfort level. Though there are few risks associated with narrowband UVB (127), there is a potential risk of skin cancer from using psoralen and ultraviolet A (PUVA) (128). The risk of skin cancer from narrowband UVB is not well established, as a systematic review found no increased risk compared with PUVA (129) and another review found insufficient evidence of risk (130). Phototherapy is not appropriate for young children, and, for all patients, the need to attend treatment sessions three times per week can impact adversely on school, work, or other commitments.

Systemic therapy using oral immunosuppressants can only be used in severe, non-responsive cases of AD. It is essential to ensure that topical therapies have been used to their maximum potential by giving comprehensive and repeated education and demonstration (103,104,131). Oral cyclosporine, azathioprine, and methotrexate are all effective systemic treatments (132–136). While the effect of cyclosporine is rapid, azathioprine- and methotrexate-induced improvements tend to emerge later. Immunosuppressant use, however, is associated with significant side effects and requires careful monitoring (133–136).

## Discussion

Despite common worldwide principles and protocols in dermatology, significant differences in global treatment methods and approaches exist. For example, systemic immunosuppressants are used more frequently in the UK and the USA than in Japan (137). Furthermore, their use differs across European countries, as reported by the European Treatment of Severe Atopic Eczema in Children Taskforce (TREAT) survey (138). Azathioprine, for instance, is used more often as a first- or second-line systemic treatment option in the UK than in other European countries, whereas oral corticosteroids are used less frequently in the UK than in Italy, the Netherlands, Spain, or Sweden. Such variations in treatment habits and approaches are not surprising in the pediatric population given the scarcity of randomized controlled AD trials and the absence of any licensed therapies.

Several new targeted approaches are emerging, which may enhance the safe and effective management of patients with AD. Dupilumab is a fully human monoclonal antibody directed against the shared IL-4 receptor α subunit that inhibits IL-4 and IL-13, which are key drivers of type 2/Th2-mediated inflammation. Dupilumab is approved for subcutaneous administration for the treatment of patients aged ≥12 years in the USA with moderate-to-severe AD inadequately controlled with topical prescription therapies or when those therapies are not advisable (139), for the treatment of adult AD patients not adequately controlled with existing therapies in Japan and for use in patients aged ≥ 12 years with moderate-to-severe AD who are candidates for systemic therapy in the European Union (140).

Through a combination of appropriate access to services, appropriate diagnosis, and appropriate use of existing approaches, we can make a significant contribution to patient benefit. However, we are entering an exciting phase of development where the number of available treatments for patients is likely to increase, offering enhanced potential to treat them safely and effectively, and to address a significant unmet need.
